# Investigating quantum metrology in noisy channels

**DOI:** 10.1038/s41598-017-16710-w

**Published:** 2017-11-30

**Authors:** B. J. Falaye, A. G. Adepoju, A. S. Aliyu, M. M. Melchor, M. S. Liman, O. J. Oluwadare, M. D. González-Ramírez, K. J. Oyewumi

**Affiliations:** 1 Department of Physics, Federal University Lafia, PMB 146 Lafia, Nigeria; 2 CIDETEC, Instituto Politécnico Nacional, UPALM, CDMX 07700 Mexico; 3 Department of Physics, Federal University Oye-Ekiti, PMB 373 Ekiti State, Nigeria; 4 Theoretical Physics Section, Department of Physics, University of Ilorin, PMB 1515 Ilorin, Nigeria

## Abstract

Quantum entanglement lies at the heart of quantum information and quantum metrology. In quantum metrology, with a colossal amount of quantum Fisher information (QFI), entangled systems can be ameliorated to be a better resource scheme. However, noisy channels affect the QFI substantially. This research work seeks to investigate how QFI of *N*-qubit Greenberger-Horne-Zeilinger (GHZ) state is affected when subjected to decoherence channels: bit-phase flip (BPF) and generalize amplitude damping (GAD) channels, which can be induced experimentally. We determine the evolution under these channels, deduce the eigenvalues, and then derive the QFI. We found that when there is no interaction with the environment, the Heisenberg limit can be achieved via rotations along the *z* direction. It has been shown that in BPF channel, the maximal mean QFI of the *N*-qubit GHZ state (${\bar{F}}_{max}$) dwindles as decoherence rate (*p*_*B*_) increases due to flow of information from the system to the environment, until *p*_*B*_ = 0.5, then revives to form a symmetric around *p*_*B*_ = 0.5. Thus, *p*_*B*_ > 0.5 leads to a situation where more noise yields more efficiency. We found that in GAD channel, at finite temperature, QFIs decay more rapidly than at zero temperature. Our results also reveal that QFI can be enhanced by adjusting the temperature of the environment.

## Introduction

Quantum entanglement and decoherence have been identified as two intertwined phenomena that form the cornerstone of quantum physics^1^. Quantum entanglement, which is the unique feature of quantum mechanics, has been a subject of active research during the past few decades^2–7^. It is not only an indispensable concept of quantum mechanics but also a new resource as real as energy^8,9^. It opens a way for conceptual understanding of classical microscopic world’s origin from the point of view of quantum mechanics. It is also an important resource for quantum metrology.

Quantum metrology plays a crucial role in science and engineering. Its applications include optical phase estimation^10^, quantum imaging^11,12^, optimal quantum clocks^13^, Quantum-enhanced positioning and clock synchronization^14^. It deals with the highest obtainable precision in various parameter estimation tasks, utilizes quantum entanglement to ameliorates the precision of parameter estimation via quantum measurements beyond the limit of its classical counterpart, and with finding measurement schemes that reach that precision^15^. The error, in this case, is limited by Cramér-Rao inequality, which is a function of Fisher information. The QFI is obtained by maximizing Fisher information over all possible positive operator value measurements. QFI is usually affected by the entanglement dynamics such as decoherence.

Decoherence is a phenomenon which arises as a consequence of the interaction between quantum systems and their environments. Naturally, closed or controlled quantum systems are difficult to find. Quantum systems unavoidably interact with the environment. This unwanted interaction results in suppression or loss of some quantum features of the quantum system^2^. For instance, the entanglement among composite system decays as it undergoes decoherence. Since this decay could hinder the development of quantum technologies, a great number of erudite scholars have developed keen interest on this subject^3,6,7^. This decay could be an asymptotic or disappearance of entanglement at a finite time (entanglement sudden death), depending upon the environment in which the quantum state is situated.

The concept of open quantum systems provides a way of exploring damping and dephasing. In most cases, the effects of such systems can be expressed via a Kraus or operator-sum representation. This representation is useful due to the fact that it provides an essential description of the principal system without considering intricate details of the environment’s properties. In this paper, we have considered two models of decoherence: the BPF and GAD channels. These two models encapsulate physics of decoherence and they can be induced experimentally. Their importance and effects have been noted down in many works of literature^16–25^.

The main objective of this paper is to scrutinize the effects of aforementioned decoherence channels on QFI of *N*-qubit GHZ maximally entangled state. A general method to deduce the maximal QFI for a given state has been investigated in ref.^26^. From a geometrical point of view, the dynamics of two variants of QFI of an arbitrary single particle state under decoherence have been reported in^27^. Within the framework of the non-Markovian dissipative process, Li *et al*.^28^ have scrutinized the dynamics of QFI of phase parameter in a driven two-level system.

It has been found that QFI gives a sufficient condition to discern multipartite entanglement. For instance, if mean QFI of a state exceeds shot-noise limit, then it’s multipartite entangled^29,30^. Within a symmetric double well, QFI has been used to distinguish and characterize behaviours of the evolved state for Bose-Einstein condensates, which shows a classical bifurcation and a transition from Josephson oscillation to self-trapping^31^. QFI has also been used to distinguish and characterize behaviours of the ground state of the Lipkin-Meskhov-Glick model. In fact, it gives a useful approach to quantum phase transition^32^.

The incessant avidity^33–40^ in studying QFI is due to the fact that it has emerged as salient quantity for quantum information theory and parameter estimation theory. QFI describes the distinctive sensitivity of a particular state with respect to perturbation of the parameter. It gives a limit to discern the family members of probability distributions. It also plays a significant role in quantum metrology^14,41^ and quantum geometry of state spaces^42^. In line with this unending interest, the current work seeks to investigate QFI of *N*-qubit GHZ entangled state in noisy channels.

## Quantum Fisher information

To deduce the precise estimation of a parameter *ϕ*, Quantum Cramér-Rao Bound (QRCB), which is a function of QFI, has been found as an indispensable tool. It adds a lower bound on the sensitivity. Basically, QFI is an extension of Fisher information within a quantum framework. The maximal mean QFI is expressed as ${\bar{F}}_{max}={C}_{max}/N$, where *C*_*max*_ denotes the largest eigenvalue of the elements in symmetric matrix given as

1 $$\begin{array}{rcl}{{\mathscr{C}}}_{mn} & = & \sum _{i\ne j}\frac{{({\lambda }_{i}-{\lambda }_{j})}^{2}}{{\lambda }_{i}+{\lambda }_{j}}[\langle {\psi }_{i}|{J}_{m}|{\psi }_{j}\rangle \langle {\psi }_{j}|{J}_{n}|{\psi }_{i}\rangle \\  &  & +\langle {\psi }_{i}|{J}_{n}|{\psi }_{j}\rangle \langle {\psi }_{j}|{J}_{m}|{\psi }_{i}\rangle ]\mathrm{.}\end{array}$$

Formulation and derivation of Eq. (1) has been shown in the Method. For a pure state, the matrix element becomes ${{\mathscr{C}}}_{mn}=2\langle {J}_{m}{J}_{n}+{J}_{n}{J}_{m}\rangle -4\langle {J}_{m}\rangle \langle {J}_{n}\rangle$. It should be noted that the condition for a state of *N* particle to be entangled is, if it cannot be written as ${\rho }_{sep}={\sum }_{k}{\otimes }_{i\mathrm{=1}}^{N}\{{p}_{k}\}|{\psi }_{k}^{(i)}\rangle \langle {\psi }_{k}^{(i)}|$ (i.e., as separable state), where {*p*_*k*_} forms a probability distribution. For a separable state, the QFI has been found to be ${F}_{Q}[{\rho }_{sep},\,{J}_{\overrightarrow{n}}]\le N$ ^29,30^.

## The noisy channels

In this section, we scrutinize the QFI for *N*-qubit GHZ state under decoherence. Two models of decoherence would be considered and these are, the BPF and GAD channels. Our assumption is that the same decoherence process affects all the qubits. The master equation, which governs the dynamics of each of these qubits, gives a map *ε* (i.e., a quantum channel which maps the input state into the output state), whose approximation can be described via Kraus representation^2,43–45^

2 $${\varepsilon }^{ch}(\rho )=\sum _{j\mathrm{=1}}^{M}{{\mathscr{K}}}_{j}^{ch}\rho {({{\mathscr{K}}}_{j}^{ch})}^{\dagger },\,{\rm{where}}\,\sum _{j}{({{\mathscr{K}}}_{j}^{ch})}^{\dagger }{{\mathscr{K}}}_{j}^{ch}=1.$$

${{\mathscr{K}}}_{j}$, which contains all the information about the dynamics of the system, are called Kraus operators. *M* denotes the number of operators needed to characterize a particular noisy channel and *ch* represents a particular noisy channel that acts on the system. Any ${{\mathscr{K}}}_{j}^{ch}$ would maps pure states onto pure states, but, a pure state will be transformed to a mixed state upon being acted on by the collective operation of several ${{\mathscr{K}}}_{j}$’s.

Evolution of *N*-qubit system can be described by Kraus operators formalism:

3 $${\varepsilon }^{ch}(\rho )=\sum _{i\mathrm{...}j}{{\mathscr{K}}}_{i1}^{ch}\otimes \ldots \otimes {{\mathscr{K}}}_{jN}^{ch}{\rho }^{N}{[{{\mathscr{K}}}_{i1}^{ch}\otimes \ldots \otimes {{\mathscr{K}}}_{jN}^{ch}]}^{\dagger },$$

where *ρ* ^*N*^ is the density matrix of the *N*-qubit system. In this study, we focus on *N*-qubit GHZ state whose density matrix can be written as

4 $${\rho }_{{\rm{GHZ}}}^{N}=\frac{1}{2}(|0\rangle {\langle 0|}^{\otimes N}+|1\rangle {\langle 1|}^{\otimes N}+|0\rangle {\langle 1|}^{\otimes N}+|1\rangle {\langle 0|}^{\otimes N})\mathrm{.}$$

### Bit-phase flip channel

The BPF is a combination of a phase flip and a bit flip. The bit flip transforms $|0\rangle$ to $|1\rangle$, and vice versa. Its operation is the same as phase damping channel. The phase flip leaves $|0\rangle$ invariant, and maps $|1\rangle$ to $-|1\rangle$, substantiating the name *phase flip*. In this subsection, we study the effect of BPF on QFI of *N*-qubit GHZ state. The general behaviours of this channel is characterized by the following Kraus operators^43–45^

5 $${{\mathscr{K}}}_{0}^{{\rm{BPF}}}=\sqrt{{p}_{B}}[\begin{array}{cc}1 & 0\\ 0 & 1\end{array}],\,{{\mathscr{K}}}_{1}^{{\rm{BPF}}}=\sqrt{{\bar{p}}_{B}}[\begin{array}{cc}0 & -i\\ i & 0\end{array}],$$

where *p*_*B*_, which appears frequently in theory of quantum error-correction, represents a certain probability with which a state is unchanged while ${\bar{p}}_{B}=1-{p}_{B}$, represents the probability of the occurrence of a BPF error. The evolution under BPF channel is given as ${ {\mathcal E} }^{{\rm{BPF}}}[{{\rm{\rho }}}_{{\rm{GHZ}}}^{(N)}]={\varrho }_{1}^{(a)}\oplus {\varrho }_{2}^{(b)}$ where

6 $$\begin{array}{rcl}{\varrho }_{1}^{(a)} & = & \sum _{\nu \mathrm{=1,}N-1}({p}_{B}^{\nu }{\bar{p}}_{B}^{N-\nu }+{p}_{B}^{N-\nu }{\bar{p}}_{B}^{\nu })|0\rangle {\langle 1|}^{\otimes \nu }|1\rangle {\langle 0|}^{\otimes (N-\nu )}\\  &  & +\sum _{\nu \mathrm{=1,}N-1}[({p}_{B}^{\nu }{(-{\bar{p}}_{B})}^{N-\nu }+{p}_{B}^{N-\nu }{(-{\bar{p}}_{B})}^{\nu })\\  &  & \times |0\rangle {\langle 0|}^{\otimes \nu }|1\rangle {\langle 1|}^{\otimes (N-\nu )}],\\ {\varrho }_{2}^{(b)} & = & \frac{1}{2}({p}_{B}^{N}+{(-{\bar{p}}_{B})}^{N})[|1\rangle {\langle 1|}^{\otimes N}+|0\rangle {\langle 0|}^{\otimes N}]\\  &  & +\frac{1}{2}({p}_{B}^{N}+{\bar{p}}_{B}^{N})[|0\rangle {\langle 1|}^{\otimes N}+|1\rangle {\langle 0|}^{\otimes N}]\mathrm{.}\end{array}$$

It can be seen that ${\varrho }_{1}^{(a)}$ is in a diagonal form already. Thus, the eigenvalues can be easily obtained as ${\bar{\lambda }}_{1}^{(a)}=\mathrm{1/2}[({(-1)}^{N-1}-1){p}_{B}{\bar{p}}_{B}^{N-1}-2{\bar{p}}_{B}{p}_{B}^{N-1}]$ and ${\bar{\lambda }}_{2}^{(a)}=\mathrm{1/2}[{(-1)}^{N-1}+1]{p}_{B}{\bar{p}}_{B}^{N-1}$ while the eigenvectors are $|{\vartheta }_{1}^{(a)}\rangle =1/\sqrt{2}({|1\rangle }^{\otimes N}-{|0\rangle }^{\otimes N})$ and $|{\vartheta }_{1}^{(b)}\rangle \mathrm{=1/}\sqrt{2}({|1\rangle }^{\otimes N}+{|0\rangle }^{\otimes N})$. By diagonalizing ${\varrho }_{2}^{(b)}$, one can find the eigenvalues as ${\bar{\lambda }}_{1}^{(b)}=\mathrm{1/2[(}-{\mathrm{1)}}^{N}-\mathrm{1]}{\bar{p}}_{B}^{N}$ and ${\bar{\lambda }}_{2}^{(b)}=1/2[({(-1)}^{N}+1){\bar{p}}_{B}^{N}+2{p}_{B}^{N}]$ and the eigenvectors as $|{\vartheta }_{1}^{(b)}\rangle =|{\vartheta }_{1}^{(a)}\rangle$ and $|{\vartheta }_{2}^{(b)}\rangle =|{\vartheta }_{2}^{(a)}\rangle$. Substituting these results into Eq. (1), we obtain the matrix element of symmetric matrix ${\mathscr{C}}$ as

7 $$\begin{array}{c}{{\mathscr{C}}}_{xx}=N({p}_{B}-{\bar{p}}_{B})({p}_{B}^{N-1}+{(-1)}^{N-1}{\bar{p}}_{B}^{N-1})={{\mathscr{C}}}_{yy}\\ {{\mathscr{C}}}_{zz}={N}^{2}({p}_{B}^{N}+{(-1)}^{N}{\bar{p}}_{B}^{N})\mathrm{.}\end{array}$$

**Figure 1:**
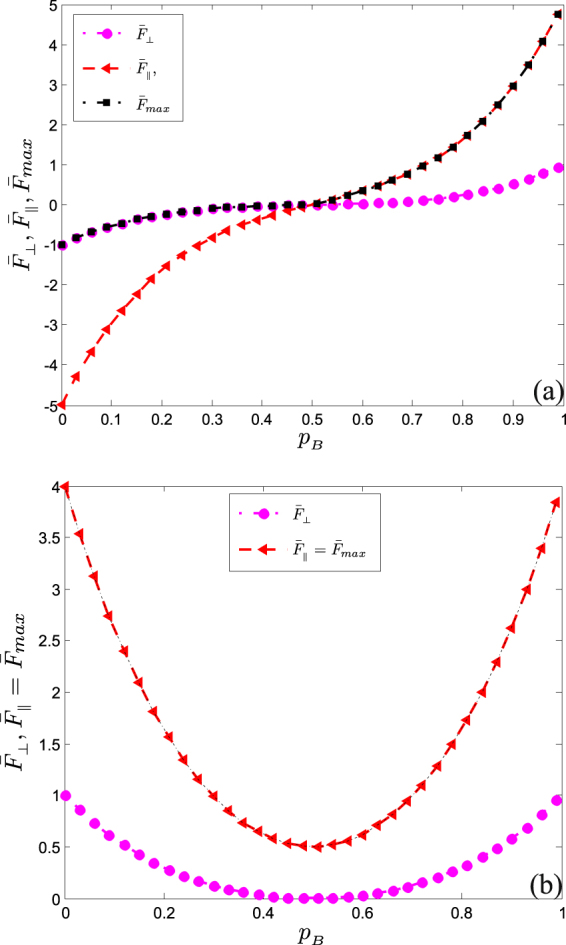
Plots of ${\bar{F}}_{max}$, ${\bar{F}}_{\perp }$ and ${\bar{F}}_{\parallel }$ as a function of decoherence rate of BPF channel. In (**a**), we consider odd *N*, i.e. *N* = 5. In (**b**), we consider even *N*, i.e. *N* = 8.

With Eq. (7), the maximal mean QFI of the *N*-qubit GHZ state can be obtain as ${\bar{F}}_{max}=1/N\times \,{\rm{\max }}\,[{{\mathscr{C}}}_{\perp },{{\mathscr{C}}}_{\parallel }]$, where ${{\mathscr{C}}}_{\perp }={{\mathscr{C}}}_{xx}={{\mathscr{C}}}_{yy}$ and ${{\mathscr{C}}}_{\parallel }={{\mathscr{C}}}_{zz}$. The subscripts _*xx*_, _*yy*_ and _*zz*_ refer to the $\overrightarrow{n}$. In Fig. 1, we examine the variation of QFI as a function of decoherence rate of BPF channel. The (−1)^*N*^ and (−1)^*N*−1^ appearing in Eq. (7), lead to disparity between odd and even *N* s. The two shapes have been depicted in Fig. 1(a) and (b). As it can be seen, when there is no interaction with the environment, the Heisenberg limit can be achieved via rotations along *z*-direction. Surprisingly, in Fig. 1(a), ${\bar{F}}_{\perp }$ and ${\bar{F}}_{||}$ increase rapidly in a concave-down manner as *p*_*B*_ increases until it varnishes at *p*_*B*_ = 0.5. For *p* > 0.5, it revives and proliferates in a concave-up manner as *p*_*B*_ increases. It can be seen from Fig. 1(b) that ${\bar{F}}_{max}$ dwindles as *p*_*B*_ increases due to flow of information from the system to the environment, until *p*_*B*_ = 0.5, then revives to form a symmetric around *p*_*B*_ = 0.5. Thus, *p*_*B*_ > 0.5 leads to a situation where more noise yields more efficiency.

### Generalized amplitude damping channel

Amplitude damping (AD) channel simulates the interaction of a quantum system with vacuum bath. It represents one of the valuable decoherence noise which provides us with description of energy-dissipation effects due to loss of energy from quantum state, for instance, the spontaneous emission of a photon or a spin system approaching equilibrium with its environment at high temperature. A simple physical model of an amplitude damping channel is the scattering of a photon through a beam-splitter. Environment represents one of the output modes which is traced out. The unitary transformation at the beam-splitter can be expressed as $B=\exp \theta ({a}^{\dagger }b-a{b}^{\dagger })$, where the creation and annihilation operators have been denoted as ${a}^{\dagger },{b}^{\dagger }$ and *a*,*b* ^20–25^. The Kraus operator for this channel can be written as ${{\mathscr{K}}}_{0}^{AD}=\sqrt{{\bar{\eta }}_{ad}}|0\rangle \langle 0|$ $+|1\rangle \langle 1|$ and ${{\mathscr{K}}}_{1}^{AD}=\sqrt{{\eta }_{ad}}|1\rangle \langle 0|$, where ${\eta }_{ad}=1-{\bar{\eta }}_{ad}\in \mathrm{[0,1]}$ denotes decay’s probability and ${\bar{\eta }}_{ad}=\exp (-{\gamma }_{0}t)$, where *γ*_0_ represents the spontaneous emission rate.

By taking the evolution of a two-level quantum system in a dissipative interaction into consideration, generalized amplitude damping channel (GAD) channel can be described by the following Lindblad form of master equation^20–25^:

8 $${\rho }^{s}(t)^{\prime} =\sum _{j\mathrm{=1}}^{2}(2{R}_{j}{\rho }^{s}{R}_{j}^{\dagger }-{R}_{j}^{\dagger }{R}_{j}{\rho }^{s}-{\rho }^{s}{R}_{j}^{\dagger }{R}_{j}),$$

where *ρ* ^*s*^ denotes reduced density matrix operator of the system interacting with a thermal bath in the weak Born-Markov rotating-wave approximation, ${R}_{1}={[{\gamma }_{0}/2({N}_{th}+\mathrm{1)}]}^{\mathrm{1/2}}R$, ${R}_{2}={({\gamma }_{0}{N}_{th}/2)}^{\mathrm{1/2}}{R}^{\dagger }$, $R=|1\rangle \langle 0|$, ${N}_{th}=1/({e}^{\hslash \omega /({k}_{\beta }T)}-1)$, ħ is reduced Planck constant, *ω* denotes the photonic’s frequency, *k*_*β*_ represents the Boltzmann constant and *T* is the temperature of the environment^20–25^.

When the environment is at zero-temperature, there would only exist loss of excitations which can be described by AD. However, practically, the environment’s temperature is always finite and to describe the dissipation effect, in this case, GAD has to be employed. To give a detail description of this system without considering the intrinsic properties of the environment, we can apply the Kraus operator-sum decomposition:

9 $${\varepsilon }^{{\rm{GAD}}}=\sum _{j\mathrm{=0}}^{3}{{\mathscr{K}}}_{j}^{{\rm{GAD}}}\rho {({{\mathscr{K}}}_{j}^{{\rm{GAD}}})}^{\dagger },$$

where the Kraus operators ${{\mathscr{K}}}_{j}^{{\rm{GAD}}}$ are,

10 $$\begin{array}{cc}{\varepsilon }_{0}^{{\rm{GAD}}}=\sqrt{p}(\begin{array}{cc}1 & 0\\ 0 & \sqrt{{\bar{\eta }}_{gad}}\end{array}), & {{\mathscr{K}}}_{1}^{{\rm{GAD}}}=\sqrt{p}(\begin{array}{cc}0 & \sqrt{{\eta }_{gad}}\\ 0 & 0\end{array}),\\ {{\mathscr{K}}}_{2}^{{\rm{GAD}}}=\sqrt{\bar{p}}(\begin{array}{cc}\sqrt{{\bar{\eta }}_{gad}} & 0\\ 0 & 1\end{array}), & {{\mathscr{K}}}_{3}^{{\rm{GAD}}}=\sqrt{\bar{p}}(\begin{array}{cc}0 & 0\\ \sqrt{{\eta }_{gad}} & 0\end{array}).\end{array}$$

$p=({N}_{th}+\mathrm{1)}/\mathrm{(2}{N}_{th}+\mathrm{1)}$ with $\mathrm{(0}\le p\le \mathrm{1)}$ is the probability of losing the excitation and also denotes the temperature of the environment. For a special case *T* = 0, then *N*_*th*_ = 0, *p* = 1 and Eqs (9) and (10) reduce to corresponding AD channel. $\bar{p}=1-p$ denotes the probability of gaining excitation^46^. The decoherence rate has been denoted by $${\eta }_{gad}=1-{\bar{\eta }}_{gad}\in [\begin{array}{cc}0 & 1\end{array}]$$ and ${\bar{\eta }}_{gad}=\exp [-{\gamma }_{0}t\mathrm{(2}{N}_{th}+\mathrm{1)]}$. Operating ${\varepsilon }^{{\rm{GAD}}}$ on the density matrix of the *N*-qubit GHZ state, yields the following evolution ${\varepsilon }^{{\rm{GAD}}}[{\rho }_{{\rm{GHZ}}}^{(N)}]={\varsigma }_{1}^{(a)}\oplus {\varsigma }_{2}^{(b)}$ where

11 $$\begin{array}{rcl}{\varsigma }_{1}^{(a)} & = & \frac{1}{2}\sum _{\nu \mathrm{=1,}N-1}[|0\rangle {\langle 0|}^{\otimes \nu }|1\rangle {\langle 1|}^{\otimes (N-\nu )}\\  &  & \times ({\eta }^{N-\nu }({p}^{\nu }{\bar{p}}^{N-\nu }+{\bar{\eta }}^{\nu }{\bar{p}}^{N})\\  &  & +{\eta }^{\nu }({p}^{\nu }{\bar{p}}^{N-\nu }+{p}^{N}{\bar{\eta }}^{N-\nu }))],\\ {\varsigma }_{2}^{(b)} & = & \frac{{{\rm{\Delta }}}_{1}}{2}|0\rangle {\langle 0|}^{\otimes N}+\frac{{{\rm{\Delta }}}_{2}}{2}|1\rangle {\langle 1|}^{\otimes N}\\  &  & +\frac{{{\rm{\Delta }}}_{3}}{2}(|1\rangle {\langle 0|}^{\otimes N}+|0\rangle {\langle 1|}^{\otimes N}),\end{array}$$

and ${{\rm{\Delta }}}_{1}={p}^{N}{\tilde{\eta }}_{gad}^{N}+{\bar{\eta }}_{gad}^{N}{\bar{p}}^{N}$ $+p{({\bar{\eta }}_{gad}\bar{p})}^{N-1}+{\bar{\eta }}_{gad}\bar{p}{p}^{N-1}$, ${{\rm{\Delta }}}_{2}={\bar{p}}^{N}{\tilde{\eta }}_{gad}^{N}+{\bar{\eta }}_{gad}^{N}{p}^{N}$ $+\bar{p}{({\bar{\eta }}_{gad}p)}^{N-1}+{\bar{\eta }}_{gad}p{\bar{p}}^{N-1}$, ${{\rm{\Delta }}}_{3}={\bar{\eta }}_{gad}^{N\mathrm{/2}}({p}^{N-1}+{\bar{p}}^{N-1})+{\bar{\eta }}_{gad}^{N\mathrm{/2}}(p{\bar{p}}^{N-1}+\bar{p}{p}^{N-1})$ and ${\tilde{\eta }}_{gad}^{N}=1+{\eta }_{gad}^{N}$. The matrix elements in Eq. (1) can be obtained via diagonalizing Eq. (11). The eigenvalues of ${\varsigma }_{1}^{(a)}$ are obtain as ${\lambda }_{1}^{(a)}=1/2{\eta }_{gad}^{N-1}(p{\bar{p}}^{N-1}+{\bar{\eta }}_{gad}{\bar{p}}^{N})$ $+\mathrm{1/2}{\eta }_{gad}(p{\bar{p}}^{N-1}+{p}^{N}{\bar{\eta }}_{gad}^{N-1})$, and ${\lambda }_{2}^{(a)}=1/2{\eta }_{gad}({p}^{N-1}\bar{p}+{\bar{\eta }}_{gad}^{N-1}{\bar{p}}^{N})$ $+\mathrm{1/2}{\eta }_{gad}^{N-1}({p}^{N-1}\bar{p}+{p}^{N}{\bar{\eta }}_{gad})$ while the eigenvectors become $|{\psi }_{1}^{(a)}\rangle ={|0\rangle }^{\otimes N}$ and $|{\psi }_{2}^{(a)}\rangle ={|1\rangle }^{\otimes N}$. For ${\varsigma }_{2}^{(b)}$, one can obtain the eigenvalues as ${\lambda }_{1}^{(b)}=1/2[{{\rm{\Delta }}}_{1}-{{\rm{\Delta }}}_{3}\,\tan (\tau \mathrm{/2})]$ and ${\lambda }_{2}^{(b)}=1/2[{{\rm{\Delta }}}_{2}+{{\rm{\Delta }}}_{3}\,\tan (\tau \mathrm{/2})]$, where $\tau =2{\tan }^{-1}[2{{\rm{\Delta }}}_{3}/({{\rm{\Delta }}}_{2}-{{\rm{\Delta }}}_{1})]$ and the corresponding eigenvectors as $|{\psi }_{1}^{(b)}\rangle =\cos (\tau \mathrm{/2)}{|0\rangle }^{\otimes N}-\sin (\tau \mathrm{/2)}{|1\rangle }^{\otimes N}$ and $|{\psi }_{2}^{(b)}\rangle =\,\cos (\tau /2){|1\rangle }^{\otimes N}$. $+\,\sin (\tau /2){|0\rangle }^{\otimes N}$ Now, by applying these results in Eq. (1), one can find

12 $$\begin{array}{rcl}{{\mathscr{C}}}_{\perp } & = & -\frac{N}{2}{({\eta }_{gad}-{\bar{\eta }}_{gad})}^{2}{(p-\bar{p})}^{2}({\eta }_{gad}^{N-1}+{\bar{\eta }}_{gad}^{N-1}\\  &  & +{p}^{N-1}+{\bar{p}}^{N-1}-2)+\frac{N}{2}\sum _{i,j\mathrm{=1}}^{2}\frac{{({\lambda }_{i}^{(b)}-{\lambda }_{j}^{(a)})}^{2}}{{\lambda }_{i}^{(b)}+{\lambda }_{j}^{(a)}},\\ {{\mathscr{C}}}_{\parallel } & = & 2\frac{{(N{{\rm{\Delta }}}_{3})}^{2}}{{{\rm{\Delta }}}_{1}+{{\rm{\Delta }}}_{2}}\mathrm{.}\end{array}$$

**Figure 2:**
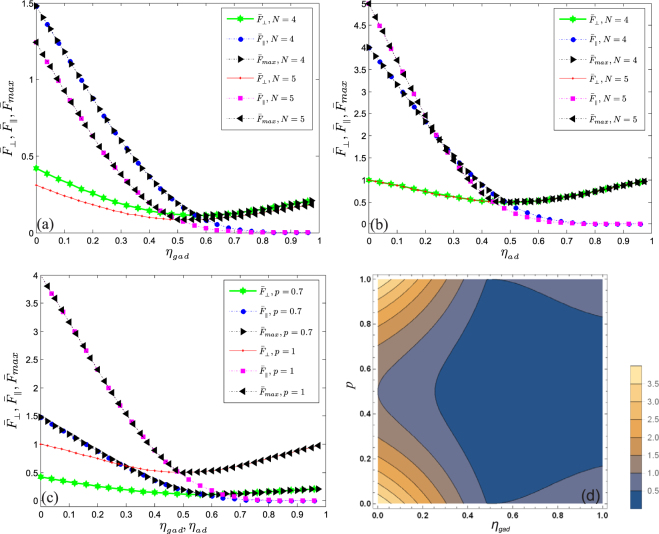
Plots of ${\bar{F}}_{max}$, ${\bar{F}}_{\perp }$ and ${\bar{F}}_{\parallel }$ as a function of decoherence rate of GAD channel (**a**) and amplitude damping channel (**b**). The initial state are 4-body and 5-body GHZ state. In (**c**), we make comparison between (**a**) and (**b**) via four-qubit GHZ state. In (**d**), we show the variation of ${\bar{F}}_{max}$ as functions of probability (*p*) and decoherence rate *η*_*gad*_ in contour plots.

In Fig. 2(a), we have plotted ${\bar{F}}_{max}$, ${\bar{F}}_{\perp }$ and ${\bar{F}}_{\parallel }$ of four- and five-qubit GHZ states as a function of decoherence rate *η*_*gad*_ with *p* = 0.7. We observe that ${\bar{F}}_{\parallel }$ decreases monotonically to zero as the strength of *η*_*gad*_ increases while ${\bar{F}}_{\perp }$ decreases gradually and then revives. The revival insinuates there is an inflow of information from the surrounding to the system. In case of five-qubit GHZ states, the vanishing characteristic of ${\bar{F}}_{\parallel }$ is discernible at ${\eta }_{gad} > 0.68$ while for four-qubit GHZ states, it is at ${\eta }_{gad} > 0.75$. This figure reveals the susceptibility of ${\bar{F}}_{max}$, ${\bar{F}}_{\perp }$ and ${\bar{F}}_{\parallel }$ to *p* and *N*. For a particular *η*_*gad*_, the enhancement becomes lager by reducing *N*. Comparing the states with higher qubit (*N* = 5) to the states with lower qubit (*N* = 4), the former are more susceptible to decoherence than the later.

In Fig. 2(b), we study the dynamics of QFI under AD channel via considering a special case *p* = 1 in Eq. (9), which corresponds to zero temperature. We observe that in the absence of noise (i.e., ${\eta }_{ad}\mathrm{=0}$), the Heisenberg limit ${\bar{F}}_{max}={\bar{F}}_{\parallel }=N$ can be achieved by considering the response of the system to SU(2) rotations in the *z* direction. It can also be seen that as the decoherence increases, ${\bar{F}}_{\parallel }$ dwindles from *N* until it varnishes. On the contrary, a shot-noise is obtained by considering the susceptibility of the GHZ state to rotations along *x* − *y* planes. As it can be seen, ${\bar{F}}_{\perp }$ decreases as *η*_*A*_ increases until ${\eta }_{A}=0.5$ where a transition occurs and then ${\bar{F}}_{\perp }$ proliferates. In accordance with the theory of quantum estimation, an increment in QFI insinuates a corresponding increase in optimal precision of estimation. From this figure, we can deduce that there exists a critical point ${{\mathscr{C}}}_{p}\approx 1-{(2N-1)}^{-\mathrm{1/}N}$. This point corresponds to where ${\bar{F}}_{max}={\bar{F}}_{\parallel }=1$.

In Fig. 2(c), we make comparison between the sensitivity of QFIs to zero temperature *N*_*th*_ = 0 and finite temperature *N*_*th*_ = 0.75. In both cases, the QFIs decreases monotonically with decoherence. This figure reveals that at finite temperature, the QFIs decay more rapidly than at zero temperature. Figure 2(d) is a contour plot which depicts the variation of ${\bar{F}}_{max}$ as functions of probability (*p*) and decoherence rate *η*_*gad*_. It is shown that the ${\bar{F}}_{max}$ can be modulated by *p*. This figure reveals the intrinsic property of GAD channel. It is shown that for ${\eta }_{gad}=0.5$, QFIs are not sensitive to any variation in the temperature of the environment.

## Discussion

Quantum metrology deals with parameter optimal estimation. Conceptual comprehension and enhancing the precision limits of quantum metrology has been of utmost necessity and it has aroused the interest of many researchers. The precision of the estimator is limited by the Cramér-Rao inequality which is a function of classical Fisher information (CFI). Maximizing the CFI over all possible measurements yields QFI. The QFI is an indispensable quantity in quantum metrology. In accordance with the theory of quantum estimation, an increment in QFI insinuates a corresponding increase in optimal precision of estimation. Unfortunately, noisy channels affect the QFI greatly. In this research work, we have studied the evolution of QFI under the influence of decoherence. Two models (i.e., BPF and GAD channels) of decoherence have been considered. The QFI has been scrutinized under these channels. We found that when there is no interaction with the environment, the Heisenberg limit can be achieved via rotations along the *z* direction. As per BPF channel, the disparity between QFI of odd and even *N* is as a consequence of (−1)^*N*^ and (−1)^*N-1*^ appearing in Eq. (7). This channel has revealed a situation whereby less noise leads to less efficiency. Also, under GAD channel, we found that QFIs are susceptible to variations in *N*. Considering a zero temperature, the GAD channel reduces to AD channel and we have examined the dynamics of QFI under this channel too. We have shown that under AD channel, by rotating the GHZ state along the *x*−*y* planes, a shot-noise can be obtained. Besides the method described in the current work (i.e. rotation of the state) to achieve a better precision, some other methods, such as optimal control^47–49^ or a carefully chosen of the initial state^50,51^ can also be used to enhance the precision of the limit.

## Method

**Figure 3:**
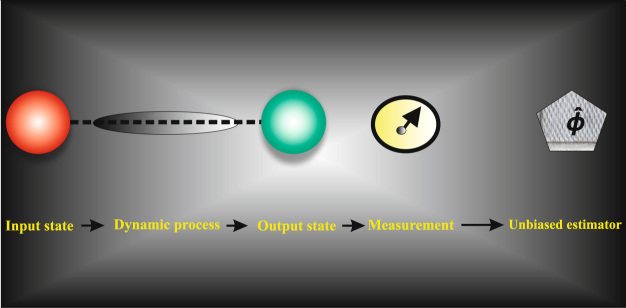
A setup for parameter estimation. A sensor prepared in a known state is sent through a $\varphi$-dependent dynamical process. The output state undergoes a measurement and from the outcome, an unbiased estimator $\hat{\varphi }$ can be produced.

There are three steps in parameter estimation: (i) preparation of the sensor’s input state (ii) the sensor undergoes a parameter-dependent dynamical process which now evolves to the final state. (iii) measurement is carried out on the final state and unbiased estimator of the parameter is roughly calculated from the result. The experimental set-up can be found in Fig. (3) below. The precision of the estimation can be determined by standard deviation, a technique of processing the data, the fluctuation of observable under consideration and the nature of the dynamic process.

Now, considering a phase shift parameter *ϕ* which can be obtained from SU(2) rotation $\rho (\varphi )={e}^{-i\varphi {J}_{\overrightarrow{n}}}\rho {e}^{+i\varphi {J}_{\overrightarrow{n}}}$ ^26^, where the operator

13 $${J}_{\vec{n}}=\vec{n}\cdot \vec{J}=\frac{1}{2}\sum _{\beta =x,y,z}{n}_{\beta }\cdot {\sigma }_{\beta },$$

represents the component of the collective angular momentum in the direction of $\vec{n}$ (which is a normalized three-dimensional vector) and *σ*_*β*_ are Pauli *x*, *y* and *z* matrices. The phase shift is considered as the value assumed by an estimator function given by $\hat{\varphi }(\{{\mu }_{i}{\}}_{m})$, where ${\{{\mu }_{i}\}}_{m}=\{{\mu }_{1},\ldots ,{\mu }_{m}\}$ represents the results of *m* independent repeated measurements (*μ*) which are described by a set of non-negative Hermitian operator ${\hat{E}}_{\mu }$ with ${\sum }_{\mu }{\hat{E}}_{\mu }=1$. The mean value and the variance of the estimator calculated over all possible sequences ${\{{\mu }_{i}\}}_{m}$ are denoted by $\langle \hat{\varphi }\rangle$ and

14 $${({\rm{\Delta }}\hat{\varphi })}^{2}=\sum _{\mu }P(\mu |\varphi ){(\varphi -\langle \hat{\varphi }\rangle )}^{2}=\langle {\varphi }^{2}\rangle -{\langle \hat{\varphi }\rangle }^{2},$$

respectively. The parameter $P(\mu |\varphi )=Tr[\rho (\varphi ){\hat{E}}_{\mu }]$ denotes conditional probability of *μ* given *ϕ*. For an unbiased estimator, $\langle \hat{\phi }\rangle =\phi$. Thus, intuitively, the explicit value of ${\sum }_{\mu }P(\mu |\varphi )(\varphi -\langle \hat{\varphi }\rangle )$ becomes 0. Moreover, differentiating this expression with respect to *ϕ*, we have

15 $$\begin{array}{c}{\partial }_{\varphi }\sum _{\mu }P(\mu |\varphi )(\varphi -\langle \hat{\varphi }\rangle )=\sum _{\mu }P(\mu |\varphi )(\varphi -\langle \hat{\varphi }\rangle )\\ \quad \quad \quad \quad \quad \quad \quad \quad \quad \,\,\,\,\oplus \times ({\partial }_{\varphi }\,\mathrm{ln}\,P(\mu |\varphi ))\mathrm{.}\end{array}$$

Using Cauchy-Bunyakovsky-Schwarz inequality “[Cauchy-Bunyakovsky-Schwarz inequality states that for all vectors **a** and **b** of an inner product space, we have | ⟨a,b⟩|2≤⟨a,a⟩⋅⟨b,b⟩|⟨a,b⟩|2≤⟨a,a⟩⋅⟨b,b⟩.]”, we have

16 $$\begin{array}{c}\sum _{\mu }P(\mu |\varphi ){(\varphi -\langle \hat{\varphi }\rangle )}^{2}\ge {(\sum _{\mu }P(\mu |\varphi ){({\partial }_{\varphi }lnP(\mu |\varphi ))}^{2})}^{-1}\\ \quad \quad \quad \quad \quad \quad \quad \quad \,\,\,\ge { {\mathcal F} }^{-1},\end{array}$$

where ${\mathcal F}$ denotes the classical Fisher information. Moreover, Eq. (16) could simply be written as ${({\rm{\Delta }}\hat{\varphi })}^{2}\ge { {\mathcal F} }^{-1}$. Also, the explicitly form of ${\mathcal F}$ becomes

17 $$ {\mathcal F} =\sum _{\mu }\frac{{[{\partial }_{\varphi }P(\mu |\varphi )]}^{2}}{P(\mu |\varphi )}=\sum _{\mu }\frac{{|Tr[{\hat{E}}_{\mu }{\partial }_{\varphi }\rho (\varphi )]|}^{2}}{Tr[{\hat{E}}_{\mu }\rho (\varphi )]}$$

Repeating the experiment *m*-times ameliorates this precision and consequently, we obtain

18 $${\rm{\Delta }}\hat{\varphi }\ge \frac{1}{\sqrt{m {\mathcal F} }}\mathrm{.}$$

The Fisher information quantifies the asymptotic usefulness of a quantum state for phase estimation. Now, let us determine the QFI (*F*) via maximizing ${\mathcal F}$ over all possible positive operator value measurements. In order to achieve this aim, it is necessary to remove ${\hat{E}}_{\mu }$ from ${\mathcal F}$ since *F* is independent of the measurement procedure. Consequently, let us consider the following definitions of logarithmic derivative *L*_*ϕ*_^52^:

1. Left Logarithmic Derivative (LLD): ${\partial }_{\varphi }\rho (\varphi )=\rho (\varphi ){L}_{\varphi }$,

2. Symmetric Logarithmic Derivative (SLD): ${\partial }_{\varphi }\rho (\varphi )=\frac{1}{2}({L}_{\varphi }\rho (\varphi )+\rho (\varphi ){L}_{\varphi })$.

Using the LLD, Eq. (17) becomes

19 $$F=\sum _{\mu }\frac{{|Tr[\sqrt{{\hat{E}}_{\mu }}\sqrt{\rho (\varphi )}{L}_{\varphi }\sqrt{\rho (\varphi )}\sqrt{{\hat{E}}_{\mu }}]|}^{2}}{Tr[{\hat{E}}_{\mu }\rho (\varphi )]},$$

where we have used the cyclic property of the trace. Now, with the help of Schwarz inequality ${|Tr[{A}^{\dagger }B]|}^{2}\le Tr[{A}^{\dagger }A]\le$, $\le Tr[{A}^{\dagger }A]Tr[{B}^{\dagger }B]$Eq. (19) becomes

20 $$\begin{array}{rcl}F & \le  & \sum _{\mu }Tr[\sqrt{{\hat{E}}_{\mu }}\sqrt{\rho (\varphi )}{L}_{\varphi }{L}_{\varphi }\sqrt{\rho (\varphi )}\sqrt{{\hat{E}}_{\mu }}]\\  & \le  & \sum _{\mu }Tr[{\hat{E}}_{\mu }\rho (\varphi ){L}_{\varphi }^{2}]\le Tr[\rho (\varphi ){L}_{\phi }^{2}]\\  & = & \frac{1}{2}Tr[{L}_{\varphi }\rho (\varphi ){L}_{\varphi }+\rho (\varphi ){L}_{\varphi }{L}_{\varphi }],\end{array}$$

which is independent of the measurement procedure ${\hat{E}}_{\mu }$ and the results *μ*. Thus, with Eqs (18) and (20), the following relation

21 $$\Delta \hat{\varphi }\ge \Delta {\varphi }_{{\rm{Q}}CR}\equiv \frac{1}{\sqrt{mF}},$$

can be established, which is the so called quantum Cramér-Rao bound (QCR). For a mixed input state, $\rho (\varphi )={\sum }_{k}{\lambda }_{k}|{\psi }_{k}\rangle \langle {\psi }_{k}|$, where ${\lambda }_{k}\ge 0$ (with ${\sum }_{k}{\lambda }_{k}=1$) denotes the eigenvalues and $|{\psi }_{k}\rangle$ represents the eigenstate. Now, in the eigenbasis of $\rho (\varphi )$, the parameter ${\partial }_{\varphi }\rho (\varphi )$ appearing in Eq. (20) becomes

22 $$\begin{array}{rcl}\langle {\psi }_{i}|{\partial }_{\varphi }\rho (\varphi )|{\psi }_{j}\rangle  & = & \frac{1}{2}(\langle {\psi }_{i}|\rho (\varphi ){L}_{\varphi }+{L}_{\varphi }\rho (\varphi )|{\psi }_{j}\rangle )\\  & = & \frac{{\lambda }_{i}+{\lambda }_{j}}{2}\langle {\psi }_{i}|{L}_{\varphi }|{\psi }_{j}\rangle \\  & = & \frac{{\lambda }_{i}+{\lambda }_{j}}{2}{L}_{{\varphi }_{ij}},\end{array}$$

where we have used SLD. Thus, *F* becomes

23 $$\begin{array}{rcl}F & = & \frac{1}{2}\sum _{i,j}\langle {\psi }_{j}|{L}_{\varphi }{\lambda }_{i}|{\psi }_{i}\rangle \langle {\psi }_{i}|{L}_{\varphi }+{\lambda }_{j}|{\psi }_{j}\rangle \langle {\psi }_{j}|{L}_{\varphi }{L}_{\varphi }|{\psi }_{j}\rangle \\  & = & \sum _{i,j}\frac{{\lambda }_{i}+{\lambda }_{j}}{2}\langle {\psi }_{j}|{L}_{\varphi }|{\psi }_{i}\rangle \langle {\psi }_{i}|{L}_{\varphi }|{\psi }_{j}\rangle \\  & = & \sum _{i,j}\frac{2{|\langle {\psi }_{i}|{\partial }_{\varphi }\rho (\varphi )|{\psi }_{j}\rangle |}^{2}}{{\lambda }_{i}+{\lambda }_{j}}\\  & = & \sum _{i\ne j}\frac{\mathrm{2(}{\lambda }_{i}-{\lambda }_{j}{)}^{2}}{{\lambda }_{i}+{\lambda }_{j}}{|\langle {\psi }_{i}|{J}_{\overrightarrow{n}}|{\psi }_{j}\rangle |}^{2}\\  & = & \overrightarrow{n}{\mathscr{C}}{\overrightarrow{n}}^{T}\mathrm{.}\end{array}$$

Now, we can write the matrix element as Eq. (1). A more rigorous form of Eq. (23) has been presented in refs^53–57^.
